# A minyan

**DOI:** 10.1017/S1478951526102879

**Published:** 2026-06-04

**Authors:** William Breitbart

**Affiliations:** Department of Psychiatry & Behavioral Sciences, Memorial Sloan Kettering Cancer Center, New York, NY, USA

She first attempted to avert my gaze, not wanting to spoil my state of innocence. With her hands and her body, my mother tried to shield from my vision other children my age, in wheelchairs, deformed by palsy and paralysis. As *a survivor* of the holocaust, she had seen too much. She did not yet want her 4-year-old son, born in the New World, to view pain, sickness, or suffering. But I was compelled to look at the faces and the eyes. Somehow, I knew we were related. This childhood experience has always been a particularly vivid and persistent memory of seemingly unknowing importance. Somehow a metaphor or an omen. As I participate in the first retreat of the *Project on Death in America’s Faculty Scholars Program* in a rustic lodge outside of Seattle, I am years and miles from the Lower East Side of this childhood memory. Yet it forces its way into my consciousness as if to say, “Look at this.”

We’ve just come from a 4-hour meeting in which the scholars shared personal experiences, which influenced us in choosing palliative medicine and care of the dying as a career path. The experience of sharing our personal stories was extremely powerful and energizing. Like the other scholars, I had prepared a “personal statement” as part of my scholar’s award application. As I came to learn at this retreat, the others had struggled endlessly with this section of the application just as I did. In fact, I had spent weeks writing the personal statement and only 2 days writing the “scientific” section of the application. You see, despite being a psychiatrist, working in a setting where caring for the dying is an integral part of my work, and being surrounded by other psychiatrists and psychologists doing similar work, I had never really been forced before to put down on paper why I chose this field of work and what it really means to me to do this work. The process was painful, private, sad, and spiritual.

We sat around a set of tables arranged in a rectangle. The sunlight, fragmented in radiating beams, lit the room in a state of calm. I looked down at my papers and scanned through my personal statement.

Personal meaning of my interest in the care of the dying: I have been working as a psychiatric clinician, researcher, and educator at Memorial Sloan-Kettering Cancer Center for 10 years. In that capacity, I have been a witness to a great deal of suffering, fear, and loss. In working with individuals and their families, confronting life-threatening cancer or AIDS, I have also witnessed enormous courage and dignity. I have spent quite a bit of time over the years trying to understand how I came to this place and this work. I believe there were 3 important influences that led me down this path.

The first influence was the holocaust. I am the firstborn son of survivors of the holocaust in Poland. My father lost his entire family with the exception of an older brother, Henry, who served in the Polish brigade of the English armed forces and fought in North Africa. After the war, Henry pursued Nazi criminals in South America. My mother was fortunate enough to have her parents and 2 brothers also survive the war. My parents married while in a displaced persons camp outside of Munich called Fahrenvalt. In 1949, they came to New York City’s Lower East Side to make a life. When I was born in 1951, my grandmother Esther wrote in Yiddish in her diary, “This is why we survived.” Throughout my adolescence, my parents and grandparents bore witness to me of the suffering, loss, courage, and dignity they had encountered in the holocaust. I was taught never to forget. I was taught compassion. I was raised to believe that I could make a difference. Growing up in this environment, I was drawn to a career that would enable me to ease suffering.

The second experience that influenced my career path was the death of my grandfather from cancer. My grandfather, Moses, was a Kohane descendant of the high priests of the Temple. I was a Levi, a descendant of those who assisted the high priests. As a Levi, I assisted my grandfather in the ritual handwashing on high holy days. I attended Sabbath services with my grandfather for 25 years until his death. I was a second-year resident in psychiatry when my grandfather was diagnosed with colon cancer. We were fortunate in that the cancer had been detected early, and a resection was possible. However, serious cardiac disease made the surgery risky, and the decision to proceed with surgery was made with trepidation. Two days before surgery, I visited my grandfather in the hospital. He seemed panicked, in a depersonalized and derealized state. He was staring into the mirror, looking pale and drawn, and asked me, “Am I alive, or am I dead?” I took my grandfather in my arms and comforted him. We sat and spoke about death for several hours. Despite his religious beliefs, he still feared death. My grandfather asked me what the experience of death would be like. He assumed that as a physician, I would know. In fact, I had seen hundreds of patients die at that point in my training. I told my grandfather what I had observed and that I felt that death could be calm and without pain. He appeared relieved. My grandfather had his surgery, but died of cardiac complications postoperatively, never having regained consciousness. The experience I had with him serves as a constant reminder of the healing that can take place in the face of death.

The third major influence was my own personal confrontation with mortality. In 1980, I was diagnosed with thyroid cancer and underwent surgery. At the time, I was a senior medical resident (having undertaken a plan to do dual residencies in psychiatry and internal medicine). During my first follow-up visit, my surgeon asked me how I was doing as he gently examined my incision site. I had been shaken by the whole experience and said, “Well, you know it’s been kinda tough.” He looked at me quizzically and said, “I don’t understand, your surgery went great.” I soon realized that while my surgeon was a technical master, he was not prepared to deal with the feelings of his patients or the emotional consequences of his surgical interventions. There was, in fact, nowhere to turn for emotional or psychological help as a cancer patient. The following year, I began to complete the remainder of my psychiatry residency. I took the opportunity to be the liaison psychiatrist for the oncology clinic. I ran groups for women with breast cancer, for the oncology clinic staff, and even conducted research. I began to read the work of 2 psychiatrists at Memorial Sloan-Kettering Cancer Center, Dr. Jimmie Holland and Dr. Mary Jane Massie. In 1984, I began a fellowship in psychiatric oncology with Drs. Holland and Massie as my mentors.

At the retreat, I was one of the first to speak. I spoke of being a child of survivors of the holocaust. I spoke of my experience with my grandfather’s death and held back the tears. Finally, I spoke of thyroid surgery. One by one, we revealed our personal stories, as if reciting a prayer. Our stories were remarkably similar. All of us, in our experiences of love, caring, and loss with our families, had discovered a respect for human life, for human relationships, and the infinite bonds that tie us all together.

As the stories were being told, I began to realize that, as a child of survivors, I had developed a strong sense of responsibility; responsibility for the vulnerable and abandoned. I had also developed a sensitivity to the emotional distress of others. Some might view these traits as burdens, and occasionally they have been. But in that room, it became clearer that they were indeed gifts. I also realized that the experience of caring for my grandfather during his last weeks of life was, in fact, a template for the work I, as a physician, would come to do with the dying. While I do not lose sight of the fact that patients I care for are not relatives or members of my family (although occasionally some do remind me of my family), I am constantly aware of my “relationship” with them, and the intimacy that develops as we look at death together.

A “minyan,” according to Jewish ritual, is the quorum of at least 10 adults required for communal worship. The concept of the minyan recognizes the critical importance of relationships to others or “connectedness” in the experiences of life, particularly in the conduct of a spiritual life and in confrontation with death. I remembered the last time I was part of a minyan. It had been many months since I had rarely attended organized religious services. A close friend of my parents, a fellow survivor, had died, and I drove my parents out to Vineland, New Jersey, for the funeral. At the gravesite, a minyan was needed to say Kaddish, the prayer for the dead. At first, I attempted to remain at the periphery of the group of mourners, but it soon became clear that I was necessary to make up this minyan. As we recited the Kaddish together, a familiar feeling began to resonate within me and envelop me. The feeling that I sensed was shared by those who stood in that circle. A feeling that was a mixture of fear, love, grief, hope, past, present, and future.

At that retreat with the other scholars, I felt once again that I was part of a minyan. Our stories, like Kaddish prayers, were both comforting and inspiring us to continue to care for the dying.

